# Prevalence and epidemiological characteristics of methicillin-resistant *Staphylococcus aureus* isolated from cattle in Bangalore India as a part of the One Health approach

**DOI:** 10.1099/acmi.0.000627.v3

**Published:** 2023-09-13

**Authors:** Nimita Venugopal, Rituparna Tewari, Feroze A. Ganaie, Susweta Mitra, Rajeswari Shome, Bibek R. Shome

**Affiliations:** ^1^​ ICAR-National Institute of Veterinary Epidemiology and Disease Informatics, Bangalore, India; ^†^​ Present address: Department of Microbiology, M.S.Ramaiah College of Arts, Science, and Commerce, Bangalore, India; ^‡^​ Present address: Centre for Cellular and Molecular Platforms, UAS-GKVK Campus, Bangalore, India; ^§^​ Present address: Department of Medicine, Division of Pulmonary, Allergy, and Critical care, University of Alabama at Birmingham, Birmingham, AL, USA; ^#^​ Present address: School of Basic and Applied Sciences, Dayananda Sagar University, Bangalore, India

**Keywords:** methicillin-resistant *Staphylococcus aureus*, multidrug resistance, multilocus sequence typing, SCC*mec* typing, *spa* typing, one health

## Abstract

In India, limited studies are available on the epidemiological aspects of methicillin-resistant *Staphylococcus aureus* (MRSA) infections in both animal and human settings. Herein, we investigated the prevalence, antimicrobial resistance profile and molecular characteristics of MRSA isolates recovered from cattle using the One Health approach. Out of 66 *mec*A-positive staphylococci, species-specific multiplex PCR detected 24 % (*n*=16) of isolates as MRSA. Maximum antibiotic resistance was seen against cloxacillin (94 %, *n*=15) and least for enrofloxacin and cephalothin (each 13 %, *n*=2). Overall, 13 % (*n*=2) of MRSA isolates were multidrug-resistant. Molecular characterization by SCC*mec* typing identified 88 % (*n*=14) of MRSA isolates as type V. Twelve isolates (75 %) belonged to novel *spa*-type t17242, of which 67 % (*n*=8) belonged to *agr* type I. MLST analysis revealed ST 1687 (50 %, *n*=8) as the most predominant sequence type. Circulation of different MRSA clones among the cattle populace offers a risk of transmission to humans through direct contact, food chain or environmental contamination. Thus, continuous monitoring of MRSA strains is imperative for early diagnosis and for establishing effective treatment strategies to restrain the disease burden caused by MRSA infections.

## Data Summary

The authors confirm that all necessary supporting data and protocols have been provided within the manuscript.

## Introduction

Methicillin-resistant *Staphylococcus aureus* (MRSA) is considered a significant threat to both human and animal health [1]. Recently, there have been growing reports of MRSA causing clinical illnesses in various animals, such as companion animals, pigs and dairy cattle [2]. The mechanism behind methicillin resistance in MRSA involves the *mecA* gene, which encodes a modified penicillin-binding protein (PBP2a) with low affinity for β-lactam antibiotics [3]. As a result, PCR-based detection of the *mecA* gene is considered the ‘gold standard’ for detecting methicillin resistance in staphylococcus [4].

Besides the *mecA* gene, there are other determinants of methicillin resistance, such as *mecC* (formerly known as LGA251), which shares 69 % nucleotide identity with *mecA*. It is believed that *mecC* MRSA emerged in animals, particularly ruminants, and subsequently spread to humans, posing a zoonotic risk [5]. Another methicillin-resistance determinant, *mecB*, has recently been discovered in *Macrococcus caseolyticus *. Given that macrococcal and staphylococcal species share the same hosts, it is likely that mobile genetic elements could facilitate the transfer of the *mecB* gene between these closely related genera [6]. Notably, methicillin-resistant *M. caseolyticus* strains from bovine and canine sources have also been found to carry a novel *mecD* gene, which confers resistance to all classes of β-lactams, including anti-MRSA cephalosporin [7].

Given the potential for MRSA to be transmitted from animals to humans, it is necessary to conduct epidemiological surveillance to track the emergence and incidence of MRSA as well as epidemic *S. aureus* clones, which may pose zoonotic risks [8]. Studies have demonstrated that methicillin-susceptible *S. aureus* (MSSA) can acquire methicillin-resistance determinants via the staphylococcal cassette chromosome *mec* (SCC*mec*) element, resulting in MRSA [9]. To better understand the epidemiological characteristics of MRSA strains, it is important to use appropriate molecular methods capable of monitoring changes over time [10–12]. Herein, we investigated the prevalence, antimicrobial resistance profile and molecular epidemiological characteristics of MRSA isolates recovered from cattle in Bangalore India using the One Health approach.

## Methods

### Sample collection and bacterial isolation

A total of 666 samples were collected from various organized (involves planned activities) and unorganized (no planned activities) cattle farms located in and around Bangalore between 2015 to 2017. The samples comprised cattle milk (*n*=371), cattle nasal swabs (*n*=109), extramammary site (*n*=90), cattle wound (*n*=30), animal handlers hand swabs (*n*=32) and environmental swabs (*n*=34). The environmental samples included feed trough (*n*=13), floor of cattle shed (*n*=15), milking machine (*n*=4) and supplied water (*n*=2). The disparity in the sample size among different sources was primarily due to resource constraints and logistical challenges.

Aseptic procedures were followed during the collection of milk samples, which involved cleaning the udder and teats with 70 % ethyl alcohol. The first few streams of milk were discarded, and 5 ml of milk was then collected in a sterile collection bottle containing 2 ml of Brain Heart Infusion (BHI) broth (Himedia Laboratories, Mumbai, India). All other samples (nasal, extramammary site, wound, animal handlers hand, environmental samples) were collected using sterile cotton swabs dipped in sterile tubes containing BHI broth. The samples were transported to the bacteriology laboratory of ICAR-NIVEDI, Bangalore, India, within 2–4 h. On arrival, the samples were immediately enriched in Mannitol salt broth (MSB) and incubated at 35–37 °C for 18–24 h. Each sample was then cultured on staphylococcus agar 110 (S110) (HiMedia, Mumbai, India) and incubated for 24 h at 37 °C. The colonies appeared as small, tiny, creamy, circular forms and some pathogenic forms also formed pigmented colonies from yellow to orange. Based on the colony morphology and pigmentation, a total of 2–3 colonies from each S110 agar plate were subsequently processed in BHI agar to yield pure cultures of isolates. The pure cultures were stored in 15 % glycerol for further downstream processing. Staphylococcus was tentatively identified based on colony morphology, pigment production, Gram staining, catalase and oxidase tests as per the standard protocol [13].

### Molecular identification of *Staphylococcus spp*

The DNA extracted from the culture isolates was subjected to multiplex PCR for the simultaneous detection of genus staphylococcus and methicillin resistance determinants (i.e. *mecA* and *mecC* genes) as described previously [14–16]. The *mecA* and/or *mecC* positive staphylococcus isolates were subjected to species-specific multiplex PCR targeting five important staphylococcus species as detailed previously [17, 18]. ATCC 33591 (*mecA* positive *S. aureus *) and BH32 (*mecC* positive *S. saprophyticus *) were used as positive control strains. BH32 strain is from the Shome Laboratory bacterial strain inventory (GenBank accession no. MG334392).

### Antibiotic susceptibility testing

Kirby–Bauer’s disc diffusion method was used to test the *mecA*-positive *S. aureus* isolates against 12 antibiotics representing five distinct antimicrobial classes (Table 1) [19]. The rationale for the choice of antibiotics examined was based on their widespread use in food animals. The isolates were classified as either susceptible, intermediate or resistant according to the guidelines of the Clinical and Laboratory Standards Institute [20]. *S. aureus* ATCC 25923 served as the quality control strain, while isolates that were non-susceptible to at least one agent in three or more antimicrobial classes were defined as multidrug-resistant (MDR) [21]. Additionally, the minimum inhibitory concentration (MIC) of cefoxitin was determined using the E-test (Himedia Laboratories). Isolates with an MIC value of ≥8 µg ml^−1^ for cefoxitin were considered methicillin-resistant [22] (Table 1). *S. aureus* ATCC 29213 was used as a quality-control strain for the MIC method.

**Table 1.** Antibiotic susceptibility profile of MRSA isolates

S. no.	Farms	Isolate ID	CX	OX	M	P	COX	CEP	AMC	AMP	E	GEN	TET	EX	CX (MIC value)
1	Farm 1	K21a	S	R	R	R	R	S	S	R	R	S	S	S	S (2)
2	Farm 1	K21b	S	R	R	S	R	S	S	S	I	S	S	S	S (0.75)
3	Farm 1	K21c	R	R	I	R	R	S	I	R	S	R	I	S	S (6)
4	Farm 1	K25a	R	R	I	R	R	R	S	R	R	I	S	S	R (>256)
5	Farm 1	K25b	S	S	S	S	S	S	S	S	S	S	S	S	S (2)
6	Farm 1	K29a	S	R	R	S	R	S	S	S	S	S	S	S	R (>32)
7	Farm 1	K52b	R	R	R	R	R	R	S	R	R	S	I	S	R (>256)
8	Farm 2	F2162My	R	R	R	R	R	S	S	R	R	R	S	S	S (6)
9	Farm 2	F2211Ww	R	R	S	R	R	S	S	R	S	R	S	S	S (6)
10	Farm 2	658 Mw	R	R	R	R	R	S	I	R	S	I	S	R	R (8)
11	Farm 2	70 Mw	S	S	S	R	R	S	S	R	S	R	S	S	S (2)
12	Farm 2	175 Mw	R	R	R	R	R	S	I	R	S	R	S	S	S (6)
13	Farm 2	328 Mw	R	R	I	R	R	S	I	R	S	R	S	S	S (4)
14	Farm 2	F2332My	R	R	R	R	R	I	S	R	I	S	I	S	R (24)
15	Farm 3	F44My	R	R	R	R	R	S	R	R	S	R	S	R	R (>32)
16	Farm 3	F41My	R	R	R	R	R	S	I	R	I	R	I	S	S (4)

Eight antibiotics viz., CX, OX, M, P, COX, CEP, AMC and AMP belong to β-lactam class of antibiotics. The other classes of antibiotics include aminoglycoside (GEN), macrolides (E), tetracycline (TET) and fluoroquinolones (EX). Isolate ID with bold letters indicate multidrug-resistant isolates, as these isolates show resistance to ≥3 classes of antibiotics.

AMC, Amoxyclavulinic acid; AMP, Ampicillin; CEP, Cephalothin; COX, Cloxacillin; CX, Cefoxitin; E, Erythromycin; EX, Enrofloxacin; GEN, Gentamycin; I, Intermediate resistance; M, Methicillin; MIC, Minimum inhibitory concentration; OX, Oxacillin; P, Penicillin; R, Resistant; S, Susceptible; TET, Tetracycline.

### Molecular epidemiological typing of methicillin-resistant *Staphylococcus aureus* isolates

The MRSA isolates were subjected to PCR-directed SCC*mec* typing as previously described by Kondo *et al*. [23] using two multiplex PCRs – one for *ccr* type assignment and another for *mec* class assignment [23]. Further, all the MRSA isolates were characterized by *spa* typing [24]. Amplification of the *spa* repeat region was performed using a set of designed primers 1067F (5′-ACGTAACGGCTTCATCCA-3′) and 1704R (5′- TCCACCAAATACAGTTGTACCG-3′). The cycling conditions involved initial denaturation at 94 °C for 5 min, 30 cycles of 94 °C for 30 s, 56 °C for 30 s, and 72 °C for 45 s followed by a final extension at 72 °C for 5 min. The PCR assay was performed in a 25 µl reaction volume containing 1×PCR buffer, 1.5U DNA *Taq* polymerase, 2 mM MgCl_2_, 200 µM deoxynucleotide triphosphates; (Fermentas, Glen Burnie, MD, USA), 0.5 µM of each primer and 50 ng template DNA. Amplified products were sequenced (Eurofins, Bangalore, India), and the resulting *spa* type and *spa* repeats were assigned by submitting the data to the *S. aureus spa* type database (http://spaserver.ridom.de/).

Multilocus sequence typing (MLST) was carried out by amplifying and sequencing seven housekeeping genes of *S. aureus *, viz., arcC, *aro*E, *glp*F, *gmk*, *pta*, *tpi* and *yqiL*. Allele number and sequence types (STs) were assigned using the *S. aureus* MLST database (https://pubmlst.org/saureus/). In addition, MRSA isolates were subjected to multiplex PCR for accessory gene regulator (*agr*) typing using *agr* group-specific primers to detect *agr* allele types (I-IV) [25]. ATCC BAA 1684 (*agr-*I), ATCC 1681 (*agr-*II), ATCC BAA 1683 (*agr*-III) were used as positive controls.

## Results and discussion

Periodic surveillance to monitor antimicrobial resistance patterns, specifically methicillin resistance in staphylococci obtained from dairy cattle could be a crucial strategy in ascertaining the emergence and spread of drug-resistant *S. aureus *. Studies have highlighted various drivers of MRSA spread within cattle-rearing settings [26]. These drivers can include factors such as intensive farming practices, inadequate biosecurity measures, overcrowding, inappropriate antibiotic use, and the proximity of livestock to humans. These factors create an environment that promotes the transmission and persistence of MRSA strains among cattle populations. Further, considering the broader One Health context, MRSA strains originating from livestock can pose a significant risk to human health [27]. Transmission of MRSA from animals to humans can occur through direct contact, occupational exposure, consumption of contaminated food products, or environmental contamination. By investigating the drivers of MRSA spread within cattle-rearing settings and their implications for human health, we can better assess the overall risk and develop appropriate cost-effective control measures. Herein, we set out to study the frequency and epidemiological features of MRSA isolates recovered from cattle in a One Health framework.

**Fig. 1.**
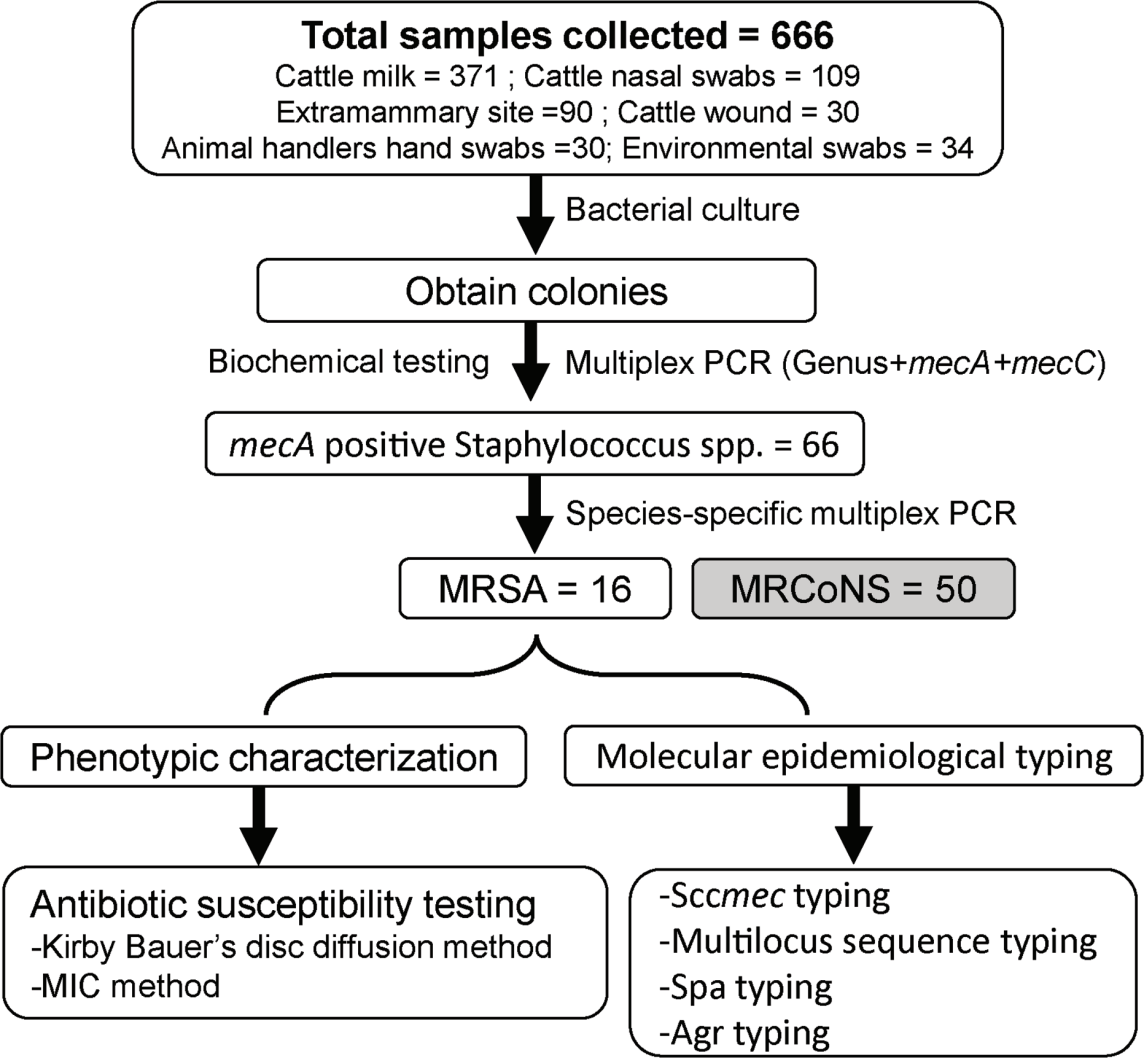
Study flow chart illustrating the events involved in the isolation and characterization of MRSA isolates. Abbreviations: MRSA, methicillin-resistant *Staphylococcus aureus *; MRCoNS, methicillin-resistant coagulase-negative staphylococcus; MIC, minimum inhibitory concentration. The multiplex PCR targets the genus *Staphylococcus* and methicillin resistance determinants (i.e. *mecA* and *mecC* genes).

As shown in Fig. 1, multiplex PCR identified a total of 66 isolates as *mecA*-positive staphylococci. None of the isolates was *mecC*-positive. Using species-specific multiplex PCR, 24 % (*n*=16) of the *mecA*-positive isolates were detected as *S. aureus* (MRSA) (95 % CI, 0.17 to 0.30), and 76 % (*n*=50) as coagulase-negative staphylococcus (MRCoNS) (95 % CI, 0.69 to 0.82). Out of the 16 MRSA isolates, 15 were recovered from cattle milk samples (95 % CI, 0.81 to 1.05), and one isolate was obtained from a cattle wound sample (95 % CI, −0.05 to 0.18). Conversely, no MRSA isolate was recovered from the other samples we collected. In European countries, the prevalence rate of bovine MRSA varies between 0.4–17 % [28, 29]. Among Asian countries, India and China report the prevalence rate of bovine MRSA to be 13 and 48 %, respectively [30, 31]. These differences in the frequencies of resistance could be attributed to variations in the animal populations examined, dissimilarity in farm management systems, different methodologies and sampling strategies implemented, variety of drug usage, and the geographical location of the studies.

While cefoxitin is a possible alternative for identifying methicillin resistance in a resource-limited situation, the usefulness of this approach is restricted by the heterogeneity in the expression of methicillin resistance in *S. aureus *. Ideally, all *mecA*-positive *S. aureus* isolates should be resistant to cefoxitin, but this is not always the case. We identified four *mecA*-positive isolates, which were sensitive to cefoxitin either by disc diffusion or MIC method. The heterogeneity may complicate the detection of MRSA, as phenotypic detection is based on various variables like temperature, pH, the osmolarity of the medium, etc. Hence detecting the *mec*A gene by PCR remains the gold standard test for the detection of MRSA. Similar observations have been reported by other groups [32–34].

Furthermore, among all the antibiotics tested, enrofloxacin was the only antibiotic to which most of the isolates (88 %) were susceptible (95 % CI, 0.72 to 0.87). In contrast, a high level of resistance was observed against cloxacillin (94 %), oxacillin (88 %), ampicillin (81 %) and penicillin G (81 %), which is in accordance with previous reports [30, 35]. The resistance pattern of MRSA isolates against other antibiotics is depicted in Table 1. The emergence of MDR MRSA is becoming a significant public health concern [36]. Overall, multidrug resistance was observed in 13 % (*n*=2) of the total MRSA isolates (95 % CI, −0.03 to 0.28) (Table 1). MIC testing of MRSA isolates against cefoxitin revealed six resistant isolates with MIC values ranging between eight to ≥256 µg ml^−1^. The rise of such strains might be attributed to the indiscriminate use of antibacterial drugs without prior drug susceptibility testing or selection pressure of antimicrobials on pathogens [37]. Consequently, the need for more effective measures against the unregulated use of antibiotics is necessary to prevent the development of MDR strains.

The majority of our MRSA isolates belonged to SCC*mec* type V (88%, *n*=14) (95 % CI, 0.71 to 1.03) (Table 2). Independent studies from Italy and Germany reported >75 % of the MRSA isolates recovered from dairy herds harboured SCC*mec* type V [38, 39]. Studies from Europe warranted that MRSA isolates with SCC*mec* type IV or V play a vital role in clinical or subclinical bovine mastitis [40]. The significance of the SCC*mec* type V in bovine MRSA has been interrelated with the resistance to zinc [41]. It is described that metal resistance genes that are frequently associated with the animal origin bacteria can promote the bacteria to fix antimicrobial resistance genes, which can be transmitted to humans through contaminated food or environment [42]. On the basis of *spa* typing, the MRSA isolates were distributed into three known *spa* types (t045, t267, and t359) and two novel *spa* types (t17242 and t18033) (Table 2). The majority (75 %, *n*=12) of the MRSA isolates belonged to t17242, with 83 % (*n*=10) of them belonging to SCC*mec* type V. Two t17242 isolates were non-typeable for SCC*mec*, which may be due to genetic variation in the target genes. Apparently, there is a high level of unpredictability in creating new SCC*mec* types in staphylococci [43], which we could not target with our assay. We found *spa* type t045 in only one MRSA isolate; whereas, a study from the US [44], found t045 in 73 % of isolates. The prevalence of *spa* types among *S. aureus* isolates varies in different areas around the world. These data are useful for defining the geographical spread of the predominant *spa* types of *S. aureus *, interpreting relative frequencies, and understanding molecular epidemiological dynamics of *S. aureus* transmission.

**Table 2.** Epidemiological typing of MRSA isolates

S. no.	Farms	Isolate ID	Source	SCC*mec* type	MLST	*Spa* type	*Agr* type
1	Farm 1	K21a	Cattle milk	Type V	ST 1687	t17242	na
2	Farm 1	K21b	Cattle milk	Type V	ST 5218	t17242	I
3	Farm 1	K21c	Cattle milk	Type V	ST 1687	t17242	I
4	Farm 1	K25a	Cattle milk	Type V	ST 5219	t18033	na
5	Farm 1	K25b	Cattle milk	Type V	ST 3881	t045	III
6	Farm 1	K29a	Cattle milk	nt	ST 1687	t17242	na
7	Farm 1	K52b	Cattle milk	nt	ST 5217	t17242	na
8	Farm 2	F2162My	Cattle milk	Type V	ST 1687	t17242	I
9	Farm 2	F2211Ww	Cattle wound	Type V	ST 5220	t17242	I
10	Farm 2	658 Mw	Cattle milk	Type V	ST 1687	t17242	na
11	Farm 2	70 Mw	Cattle milk	Type V	ST 5216	t359	I
12	Farm 2	175 Mw	Cattle milk	Type V	ST 1687	t17242	I
13	Farm 2	328 Mw	Cattle milk	Type V	ST 1687	t17242	I
14	Farm 2	F2332My	Cattle milk	Type V	ST 5217	t267	I
15	Farm 3	F44My	Cattle milk	Type V	ST 2668	t17242	I
16	Farm 3	F41My	Cattle milk	Type V	ST 1687	t17242	I

na, not available; nt, Non-typeable.

**Fig. 2.**
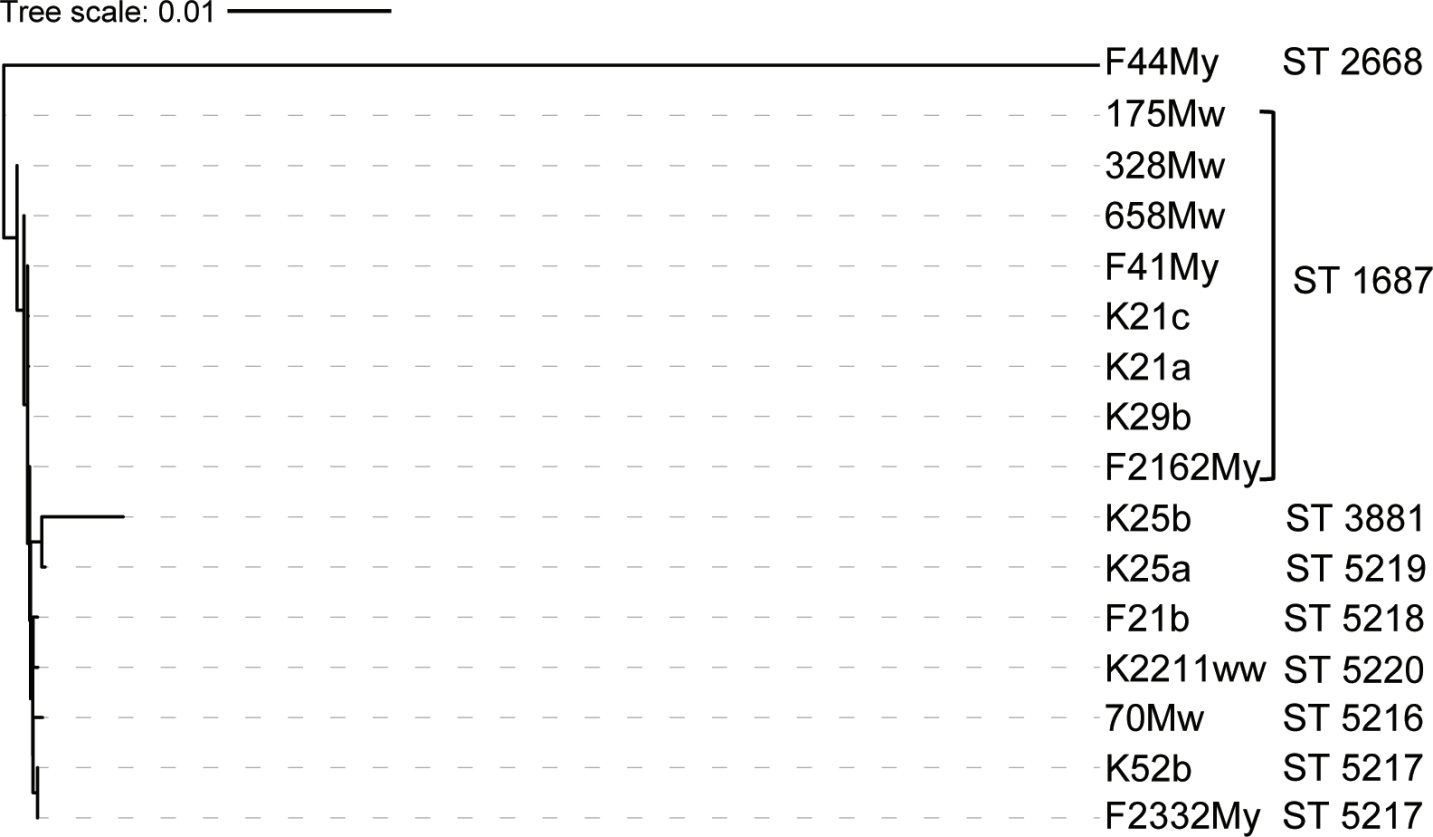
Phylogenetic analysis of MRSA isolates. MRSA strains were distributed into different clades based on the phylogeny of concatenated MLST sequences (seven housekeeping MLST genes). The length of the scale bar represents the estimated evolutionary divergence between strains based on average genetic distance. A scale bar of 0.1 indicates 10 % nucleotide substitution per site. The phylogenetic tree was built using Geneious prime version 2021.0.3 and iTOL version 6.8 was used for visualization.

Moreover, MLST and *agr* typing represent the key markers to study the epidemiology of *S. aureus *. MLST analysis revealed ST 1687 (50 %, *n*=8) as the most common ST among MRSA isolates (95 % CI, 0.37 to 0.62), with seven of them belonging to *spa* type t17242 and SCC*mec* type V. In addition, five novel STs were detected viz., ST 5217 (*n*=2) and one each of ST 5216, ST 5218, ST 5219 and ST 5220 (Table 2). Interestingly, bovine MRSA isolates belonging to a novel ST 1687 were first reported from India [45]. Phylogenetic analysis of MRSA isolates (Fig. 2) revealed that ST 2668 exhibited the maximum divergence in the population framework. The findings highlight the potential significance of ST 2668 as a major source of spread within the study site.

*S. aureus* derived from hospitals and cattle was shown to have a high prevalence of *agr* type I [46, 47]. We detected *agr* type I in 63 % (*n*=10) of MRSA isolates (95 % CI, 0.45 to 0.79), which is in accordance with earlier reports [48, 49], indicating *agr* type I as the frequent group among the *S. aureus* isolates. Moreover, 67 % (*n*=8) of *spa* type t17242 belonged to *agr* type I. Ten isolates with *agr* type I were distributed into six STs and three *spa* types (ST 5218–t17242, ST 1687–t17242, ST 5220–t17242, ST 2668–t17242, ST 5216–t359 and ST 5217–t267), with the most predominant clone being ST 1687– t17242–*agr* type I. Studies from South Korea by Lim *et al*. detected ST 72–t324–SCC*mec* type IV as the most predominant clone in cow milk, farmers and farm environment [50]. In India, the predominant clone of MRSA causing bovine mastitis in regions of Telangana, Tamil Nadu and Andhra Pradesh was found to be ST 72–t17287, with the majority of the isolates carrying SCC*mec* type III [45]. Previous studies have shown that *S. aureus* strains belonging to *agr* group I can invade epithelial cells of the mammary gland, demonstrating that *agr* type I strains can be the major etiological agent in mastitis [51].

## Conclusion

The overall prevalence of MRSA is high among methicillin-resistant staphylococci in dairy cattle. The grave concern is the emergence of MDR MRSA strains, which pose a potential risk to animal and public health at large. Since MRSA transmission is dynamic and can empirically traverse any animal–human interface, continuous monitoring of drug resistance in *S. aureus* is critical to developing effective treatment strategies and preventing the disease burden caused by MRSA infections.
